# Procyanidin C1 Location, Interaction, and Aggregation in Two Complex Biomembranes

**DOI:** 10.3390/membranes12070692

**Published:** 2022-07-05

**Authors:** José Villalaín

**Affiliations:** Institute of Research, Development, and Innovation in Healthcare Biotechnology (IDiBE), Universidad Miguel Hernández, E-03202 Elche, Spain; jvillalain@umh.es; Tel.: +34-966-658-762; Fax: +34-966-658-758

**Keywords:** procyanidin C1, plasma membrane, mitochondrial membrane, molecular dynamics

## Abstract

Procyanidins are known for their many benefits to human health and show a plethora of biological effects. One of the most important procyanidin is the procyanidin trimer C1 (PC1). Due to its relatively high lipid–water partition coefficient, the properties of PC1 could be attributed to its capability to interact with the biomembrane, to modulate its structure and dynamics, and to interact with lipids and proteins, however, its biological mechanism is not known. We have used all-atom molecular dynamics in order to determine the position of PC1 in complex membranes and the presence of its specific interactions with membrane lipids, having simulated a membrane mimicking the plasma membrane and another mimicking the mitochondrial membrane. PC1 has a tendency to be located at the membrane interphase, with part of the molecule exposed to the water solvent and part of it reaching the first carbons of the hydrocarbon chains. It has no preferred orientation, and it completely excludes the CHOL molecule. Remarkably, PC1 has a tendency to spontaneously aggregate, forming high-order oligomers. These data suggest that its bioactive properties could be attributed to its membranotropic effects, which therefore supports the development of these molecules as therapeutic molecules, which would open new opportunities for future medical advances.

## 1. Introduction

Procyanidins are a group of bioactive molecules that are known for their benefits to human health. The use of these molecules is increasing in the treatment of different chronic diseases, such as cancer, diabetes, and cardiovascular diseases, since they supposedly prevent cell damage that is possibly related to oxidative stress. Procyanidins are included within the condensed tannins group and, together with the hydrolysable tannins groups, form the tannin group of heterogeneous phytochemicals with a high molecular weight [1,2]. Tannins, in general, form a defense strategy in plants against both biotic and abiotic stresses [1]. Procyanidins belong to the polyphenols group, which comprise one of the most dominant classes of antioxidant bioactive compounds in nature [3]. Interestingly, the quantity of procyanidins is very high in vegetables, fruits, cereals, and the seeds of numerous species of plants [2,4] and they are very heterogeneous compounds with a diverse chemical structure [5,6]. Most importantly, they have been shown to possess a plethora of valuable properties, which include anticarcinogenic, antimicrobial, antiviral, antiinflammatory, antiallergic, antimutagenic, and antihyperglycaemic effects [3,7,8,9,10,11,12,13,14,15].

**Figure 1:**
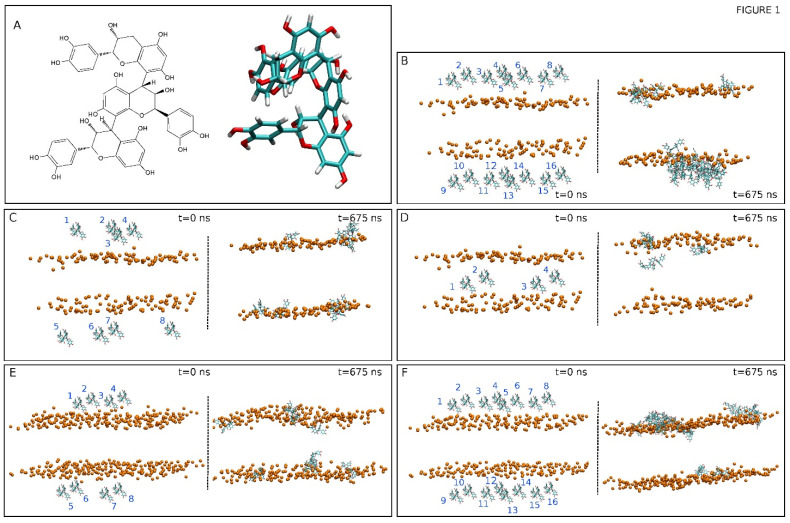
(**A**) Chemical and molecular structures of procyanidin C1, PC1 [(-)-epicatechin –(4β→8)-(-)-epicatechin-(4β→8)-(-)-epicatechin]. The initial, t = 0 ns, and final, t = 675 ns, systems for (**B**) system 1, (**C**) system 2, (**D**) system 3, (**E**) system 4, and (**F**) system 5. The disposition of the PC1 molecules (numbered) in each one of the systems are also displayed. The PC1 molecules are depicted in licorice representation and the phosphate atoms of the phospholipids, defining the upper and lower boundaries of the membrane, are depicted in VDW representation and an orange colour. The lipid and water molecules and the chloride and sodium ions have been removed for clarity.

The procyanidin trimer C1 (PC1) is one of the most important procyanidins with many biological effects (Figure 1A). For example, PC1 has antiproliferative properties as it can induce apoptosis in breast cancer cells [16]; in addition, it also prevents melanoma cell growth [17]. PC1 has also been shown to inhibit the Na^+^,K^+^-ATPase [18] and the H^+^,K^+^-ATPase [19] and it has been demonstrated that PC1 also promotes preadipocyte differentiation and enhances insulin sensitivity [20,21]. Moreover, PC1 has senotherapeutic activity and increases the health and the life span of mice, possibly through the impairment of the functional integrity of the mitochondria [22]. Due to its relatively high lipid–water partition coefficient, the possible effects of PC1 on the biological systems could be attributed to its capability to locate the biological membrane and to modulate the membrane structure and dynamics. The interaction of flavonoids in general (procyanidins in particular) with the membrane components, in both proteins and lipids, might be responsible, at least in part, for their effects on human and animal health [23]. The XLogP3 value [24] of PC1 is 3.3, but its hydrophobic properties do not give us any indication about its location, orientation, or its possible interactions with the membrane. Significantly, it has been shown that procyanidins can interact with membrane lipids, which alters the biophysical properties of biomembranes [25,26]. Specifically, hexameric procyanidins can interact with membrane lipid rafts [27], which could imply that other procyanidins (shorter and larger oligomers) could also interact with lipid rafts, either sphingomyelin, cholesterol, or both, as well as with other types of membrane lipids. PC1 can distress the biological membrane, interact with the cellular proteins, or both, however, due to its high number of beneficial and different biological effects, a common mechanism should be at play. It could, therefore, be thought that its mechanism of action could be ascribed to its capability to interact with the biological membrane, modulate its fluidity, morphology, and permeability, and, finally, to interact with its components. The knowledge of the PC1 mechanism of action might open up new avenues for future therapeutic developments of this molecule or other related molecules. It is important to note that, under gastric conditions, procyanidins dimers and trimers do not transform into their monomer constituents, and they are found intact in the plasma after ingestion [28,29]. Moreover, high-concentration and high-frequency treatment of procyanidin C1 has no systemic toxicities [22,30].

Molecular dynamics (MD) is perfectly suitable for learning about the dynamics, the location, the interaction, and the structure of many different types of molecules interacting with biological model membranes [31,32]. We have used atomistic MD to define the location and the orientation of PC1 in the membrane and, at the same time, to determine the presence of any interactions with the membrane lipids. It is difficult to attempt to simulate a system as complex as that of a biological membrane. Furthermore, one has to consider the existence of different biological membranes, due to their different lipid and protein composition. Since the biological effects of PC1 could be related to the cellular oxidative stress, we have studied two different model biomembrane systems; one of them was similar to the lipid composition of the plasma membrane (PM) and the other was similar to the mitochondrial membrane (MIT). We believe that this approximation is a valid one and, therefore, the results obtained could be extrapolated to the situation that occurs in a biological cell; that is, to explain the possible mechanism of action of the PC1 molecule at the membrane level. For that goal, we have studied five different systems, three PM-derived and two MIT-derived, containing different numbers and locations of PC1 molecules (Table 1). Our results suggest that PC1 tends to be primarily placed at the membrane interface, between the phosphate atoms of the phospholipids and the oxygen atom of CHOL, which have a high propensity to aggregate through hydrogen bonding with each other which therefore affects the biophysical properties of the membrane lipids. Our data suggest that the bioactive properties of PC1 could be attributed to its membranotropic effects and accordingly through the modulation of the biophysical membrane properties.

## 2. Materials and Methods

Unrestrained all-atom MD simulations were carried out using NAMD 2.14 [33] with the CHARMM36 protein and lipid force fields [34,35,36]. All MD parameters used in this work have been described previously [37,38]. The whole systems, comprising water, ions, membrane, and protein/s, were equilibrated before the simulation for 5 ns after 100,000 steps of minimization, so as to remove unfavorable atomic contacts. The production trajectory for each one of the five protein/membrane systems studied in this work was calculated for a total of 675 ns (Table 1).

We have studied two different model biomembrane systems, one of them was similar to the lipid composition of the plasma membrane (PM) and the other was similar to the mitochondrial membrane (MIT). We have studied five systems, three PM-derived and two MIT-derived, each one containing a different number of PC1 molecules and locations (Table 1). The membrane systems were assembled using the Charmm-Gui web server [39]. The systems contained excess water [40]. Each one of the systems was composed of PC1 molecules, a membrane bilayer, water, and NaCl at physiological conditions, i.e., a concentration of 0.15 M, enclosed in a rectangular box, and in a neutral setting (Table 1) [41,42,43]. The PM system contained 80 molecules of 1-palmitoyl-2-oleoyl-sn-glycero-3-phosphocholine (POPC), 48 molecules of 1-palmitoyl-2-oleoyl-sn-glycero-3-phosphoethanolamine (POPE), 18 molecules of 1-palmitoyl-2-oleoyl-sn-glycero-3-phosphoserine (POPS), 16 molecules of 1-palmitoyl-2-oleoyl-sn-glycero-3-phosphoinositol-3-phosphate (PI-3P), 34 molecules of N-stearoyl-D-erythro-sphingosylphosphorylcholine (PSM), and 84 molecules of cholesterol (CHOL). The MIT system contained 90 molecules of POPC, 68 molecules of POPE, 2 molecules of POPS, 10 molecules of PI-3P, 2 molecules of PSM, 2 molecules of 1-palmitoyl-2-oleoyl-sn-glycero-3-phosphate (POPA), 28 molecules of 1′,3′-bis [1-palmitoyl-2-oleoyl-sn-glycero-3-phospho]-glycerol (cardiolipin, CL), and 20 molecules of CHOL [44,45,46] (Table 1). The chemical structures of the lipid molecules used in this study are shown in Supplementary Figure S1. The presence of one oleoyl chain in the phospholipids increase its fluidity in the membrane, makes the membrane more similar to the real one, and reduces the simulation time. The total number of lipids was 280, 140 per leaflet, for the PM system and 222, 111 per leaflet for the MIT system. The normal bilayer was parallel to the *z*-axis of the membrane and its surface constituted the x–y plane. The height of the simulation box and the cross-sectional area were permitted to fluctuate independently of each other with no constraints. The molecule of PC1 was created and minimized using Discovery Studio 4.0 (Accelrys Inc., San Diego, USA). The CHARMM General Force Field (CGenFF) compatible stream files of PC1 were obtained using the Charmm-Gui web server [39]. The initial arrangements at t = 0 ns are depicted in Figure 1B–F for all five different systems. Systems 1 to 3 contained the PM membrane and systems 4 and 5 contained the MIT membrane. System 1 contained 16 molecules of PC1 at the external part of the membrane, with 8 at each side of the membrane (Figure 1B). System 2 contained 8 molecules of PC1 at the external part of the membrane, with 4 at each side of the membrane (Figure 1C). System 3 contained 4 molecules of PC1 at the middle of the palisade part of the membrane (Figure 1D). System 4 contained 8 molecules of PC1 at the external part of the membrane, with 4 at each side of the membrane (Figure 1E). System 5 contained 16 molecules of PC1 at the external part of the membrane, with 8 at each side of the membrane (Figure 1F). The distance between the PC1 molecules in water and the membrane surface (represented by the phosphate atoms of the phospholipids) was 16 Å.

The average layer of the lipid head-group phosphate atoms defined the membrane surface and were parallel to the x–y plane. VMD software was used for analysis and visualization, as before [37,38,47,48]. The center-of-masses and number of molecular contacts were obtained using standard VMD plugins. S_CD_ order parameters, surface area per lipid, membrane thickness, molecular areas, and molecule tilt were calculated as previously described [49] using VMD “Membplugin” [48]. Mass density profiles were obtained by means of the VMD “Density Profile Tool” plugin [50]. The presence of hydrogen bonds were defined by a distance of less than 3 Å between the acceptor and donor atoms and an acceptor-H-donor angle of at least 150° [51]. The complete simulations were used for analysis, i.e., 675 ns, unless otherwise stated.

## 3. Results

We have used five different membrane/PC1 systems, each one of them divergent from the others by the number and the location of the PC1 molecules in the system, as well as in the model biomembrane used (Table 1). System one contained the PM membrane and 16 molecules of PC1 at the external part (Figure 1B), system two contained the PM membrane and eight molecules of PC1 at the external part (Figure 1C), system three contained the PM membrane and four molecules of PC1 at the middle part of the bilayer (Figure 1D), system four contained the MIT membrane and eight molecules of PC1 at the external part of the membrane, with four at each side of the membrane (Figure 1E), and system five contained the MIT membrane and 16 molecules of PC1 at the external part of the membrane (Figure 1F). The time variation of the lipid area, as well the time variation of the membrane thickness, were used in order to assess the membrane system equilibration both during and at the end of the simulation [52,53]. The lipids areas remained constant after ~35 ns (Supplementary Figure S2). The mean area of all of the lipids in the different systems for the last 30 ns of simulation are shown in Supplementary Table S1. The obtained data show that average molecular areas were comparable to those that have been previously reported [49,54,55]. The membrane thickness also remained practically constant after ~35 ns (Supplementary Figure S3A). The average membrane thickness for all of the systems for the last 30 ns was 43–44 Å (rounded to the first integer, Supplementary Table S2 and Supplementary Figure S3B), which is comparable to those that have been described for systems containing CHOL [54]. This data indicate that the systems were equilibrated early on in the progress of the simulation and have revealed that the membrane systems attained a steady state after ~35 ns of the simulation.

At first, the PC1 molecules were placed in both the upper and lower water layers and, in the case of system three, in the middle of the membrane (see Figure 1). The final, t = 675 ns, snapshots for all of the systems are shown in Figure 1B–F. At the end of the simulation, the PC1 molecules, which at the beginning of the simulation were located in the middle of the upper and lower water layers, had moved to a position near the membrane interface (systems one and two for the PM membrane system, Figure 1B,C, and systems four and five for the MIT membrane system, Figure 1E,F). In a similar way, the PC1 molecules, which at the beginning of the simulation were inserted into the middle of the membrane, moved to a place near to the membrane interface, except for one system (system three, Figure 1D). However, interestingly, many of the PC1 molecules, depending on the system, aggregated with time. Not only did they form dimers, trimers, or tetramers, but also higher-order oligomers (Table 2). In system one, which had 16 PC1 molecules, only two of them were in the monomer state, four of them were in the dimer state, and ten of the molecules formed an oligomer (Table 2). This is in contrast to system two, which had half of the concentration of system one, i.e., eight PC1 molecules, which had five PC1 molecules in the monomer state and only three forming a trimer (Table 2). System four, which had eight PC1 molecules, had six PC1 molecules in the monomer state and two forming a dimer (Table 2). On the other hand, system five, which had 16 PC1 molecules, had four monomers, one tetramer, and an oligomer that was formed by eight molecules of PC1 (Table 2). System three was different from the others in the sense that it only had four molecules of PC1 inserted into the middle part of the membrane. In this case, all of the molecules were in the monomer state at the termination of the MD simulation (Table 2). From these data, it is clear that PC1 aggregates in water in a concentration-dependent mode and it is independent of the model membrane system. The total number of monomers, taking into account all of the five systems, was 21 and the number of PC1 molecules in aggregated form was 31 (Table 2). We have obtained the number of contacts and hydrogen bonds between the pairs of PC1 molecules for the last 30 ns of the MS simulation, either forming dimers or dimers in higher-number oligomers. The average number of contacts between each pair was 15.7 ± 5.9 and the average number of hydrogen bonds was 0.96 ± 0.66. This data highlights the predisposition of PC1 molecules to aggregate both in solution and at the membrane surface.

**Figure 2:**
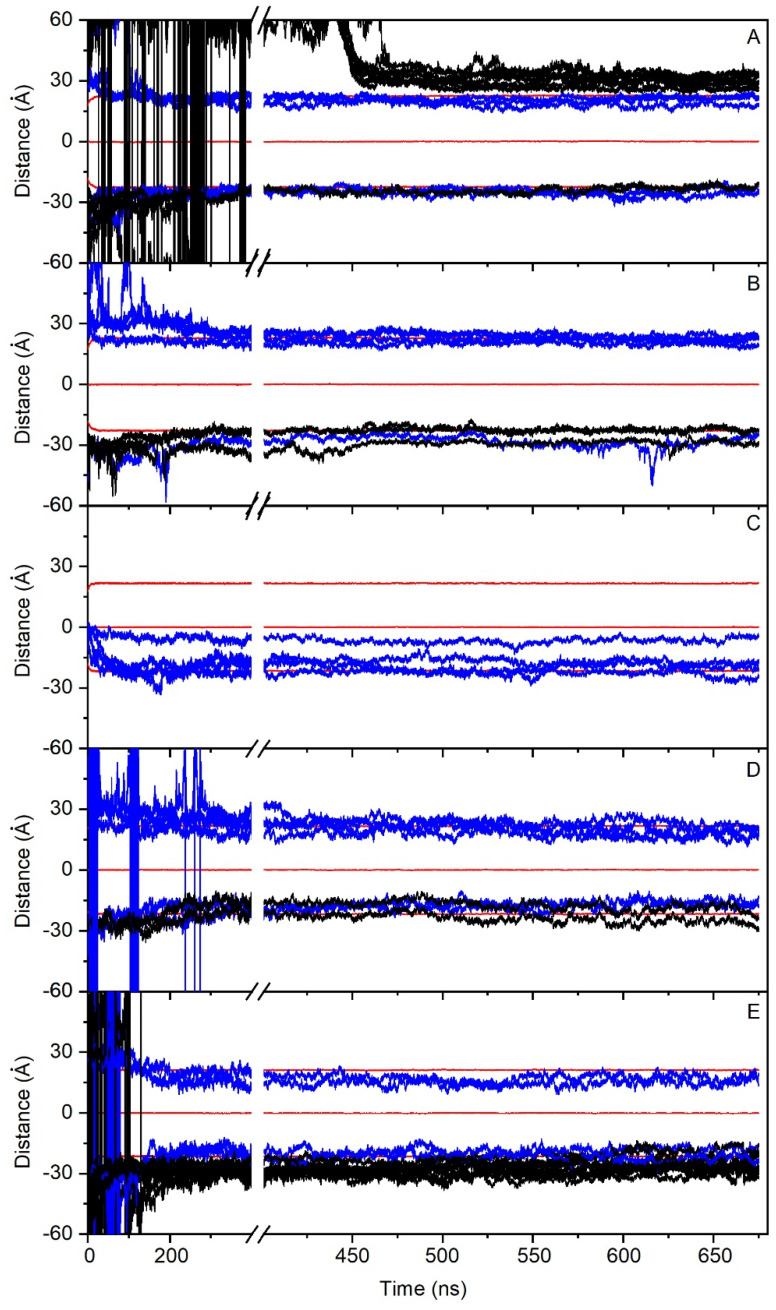
Time variation of the *z*-axis distance (middle of the membrane as a reference) for the different PC1 molecules in (**A**) system 1 (16 molecules), (**B**) system 2 (8 molecules), (**C**) system 3 (4 molecules), (**D**) system 4 (8 molecules), and (**E**) system 5 (16 molecules). PC1 molecules in the monomer state are depicted in blue, whereas the PC1 molecules forming dimers or higher-state oligomerization are depicted in black. The *z*-axis distance of the phosphate atoms of the phospholipids, defining the membrane surface, is depicted in red (upper, center, and lower boundaries). See text for details.

In order to check the conduct of all of the PC1 molecules in the membrane throughout the whole simulation, we have obtained the time variation of their center-of-mass (COM) compared with the COMs of the phosphate atoms in both leaflets, which make up the membrane surface (Figure 2). For system one, eight molecules of PC1 were located at the middle of the upper water layer and eight molecules of PC1 were located at the middle of the lower water layer (Figure 1B). At the beginning of the MD simulation, some of them displayed a significant fluctuation in their COM, whereas others did not (Figure 2A). Interestingly, and at around 400–450 ns, several of the freely moving PC1 molecules in the water layer dramatically reduced their motion, coinciding with the time when those PC1 molecules formed a massive aggregate (Figure 2A). The histograms corresponding to the COM of all of the PC1 molecules in system one and for the last 30 ns of the simulation are shown in Supplementary Figure S4, where it can be observed that all of them were close to the membrane surface and some had even crossed it. Interestingly, of the sixteen different isolated molecules at the beginning of the simulation, only two remained in the monomer state (Table 2). In the case of system two, four molecules of PC1 were located at the middle of the upper water layer and four molecules of PC1 were located at the middle of the lower water layer (Figure 1C). As observed in Figure 2B, and at the beginning of the MD simulation, some of them displayed a significant oscillation in their COM. However, at an early stage of less than 200 ns, they moved to a position near to the membrane surface and their erratic movement stopped (Figure 2B). In this case, only one trimer was encountered, the other PC1 molecules remained in the monomer state throughout the MD simulation (Table 2). The histograms corresponding to the COM of all of the PC1 molecules and for the last 30 ns of the simulation is shown in Supplementary Figure S5, where it can be observed that all of them were close to the membrane surface and some had even crossed it. System three was very different to the previous systems, since four molecules of PC1 were located at the middle of the membrane bilayer (Figure 1D). At the beginning of the MD simulation, all of them displayed a significant fluctuation in their COM, but after about 100 ns of the simulation time they did not move significantly from the position until the end (Figure 2C). All four of the molecules remained in the monomer state for all of the MD simulation (Table 2), three of them located to a position near to the membrane surface, but one PC1 molecule remained at the hydrocarbon region of the membrane. This tendency can be observed in the histograms corresponding to the COMs of the last 30 ns of the simulation (Supplementary Figure S6). Systems one to three comprised the PM model membrane. However, systems four and five comprised the MIT model membrane. System four had eight molecules of PC1, of which four were in the middle of the upper water layer, and four were in the middle of the lower water layer (Figure 1E). At the beginning of the MD simulation, and similar to the other systems, many of them displayed a significant fluctuation in their COM, whereas other did not (Figure 2C). However, after about 300 ns, the movement on the *z*-axis of all of the PC1 molecules stopped since all of them were located near to the surface of the membrane (Figure 2C). For system four, only one dimer was formed, the other six molecules remained in the monomer state (Table 2). The histograms for the last 30 ns of the MD simulation show the location of the PC1 molecules in this system (Supplementary Figure S7). System five had 16 molecules of PC1 in total, of which eight molecules were located at the middle of the upper water layer and eight molecules were located at the middle of the lower water layer (Figure 1F). Similar to the other systems, and at the beginning, all of them had an erratic system at the water layer; however, at about 150 ns they were approaching the membrane surface and remained there, their movement restricted to the layer near to it (Figure 2E). For this system, only four molecules remained in the monomer state, while the others formed a tetramer and an octamer (Table 2). Supplementary Figure S8 shows the histograms of the COMs for the last 30 ns of the simulation. It is possible to observe that all of the PC1 molecules, either forming monomers or oligomers, were located near to the membrane surface.

**Figure 3:**
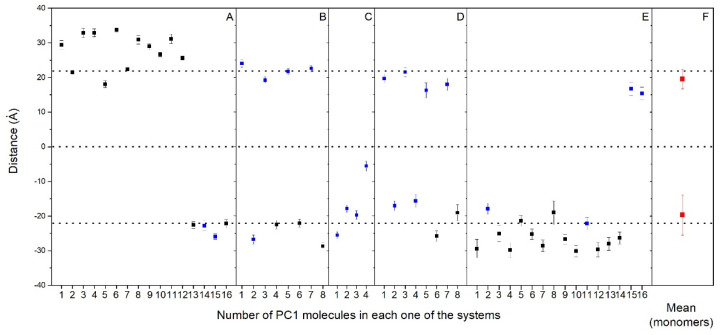
Average *z*-axis distance (middle of the membrane as a reference) for the different PC1 molecules and for the last 30 ns of the MD simulation in (**A**) system 1, (**B**) system 2, (**C**) system 3, (**D**) system 4, and (**E**) system 5. PC1 molecules in monomer (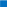) and oligomerized (■) states are shown. The mean plus standard deviation for all the PC1 molecules in the monomer state (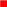) is shown in (**F**). The dotted lines define the membrane surfaces and the center of the bilayer (phosphate atoms of the phospholipids).

As observed in Figure 2 and Supplementary Figures S4–S8, nearly all of the PC1 molecules at the conclusion of the simulation time were located near to or at the membrane surface, either with the PM model membrane (systems one to three) or the MIT model membrane (systems four and five). It is very interesting to compare the location of all of the PC1 molecules in all of the systems studied, as shown in Figure 3, where the mean COMs for the last 30 ns of the simulation are shown. All of them, even the aggregated ones, are all less than 10 Å from the surface, except for one PC1 molecule in system three, which is near to the center of the bilayer (Figure 3). If we take only the location data of the PC1 molecules in the monomer state, the mean COM for the positive layer was 19.5 ± 2.8 Å, whereas the mean COM for the negative layer was −19.7 ± 5.8 Å (Figure 3F), i.e., an absolute global mean COM of 19.6 Å. Taking into account that the positive membrane surface was 22.1 ± 0.5 Å and the negative membrane surface was −22 ± 0.6 Å, the preferred location of the PC1 molecules is one in which the COM is about 2.5 Å below the membrane surface (Figure 3). However, we are talking about the COM of the molecule, but we have to take into account the real space that the PC1 molecule occupies. The molecule is, approximately, a rectangle of dimensions of about 15 Å·12 Å·12 Å, so that the molecule tends to be positioned in the membrane, with part of it extending 4–5 Å outside and 8–10 Å inside of it. The average mass density of all of the components for the last 30 ns of the biomembrane model systems are presented in Supplementary Figure S9. All of the profiles are practically symmetric between the two leaflets of the membrane, which implies a comparable behavior for all of the lipids inside of them. By comparing the mass density profiles between both the PM and the MIT systems (Supplementary Figure S9A–E,G–I, respectively), it is interesting to point out that the profiles are very similar, indicating a similar behavior of all of the lipids in both of the systems. As observed in the Figure 3, the global mass density of all of the PC1 molecules extends to the external part of the membrane, as well as to the internal part, passing the phosphate atom layer but never extending beyond the molecules of cholesterol. If we observe the mass density profile of each individual PC1 molecule compared to the phosphate mass density, this pattern looks much better in the sense that their location is more defined. Comparing the location of the PC1 molecules in the monomeric state with those that form aggregates, it can be clearly seen that all of them, whether in the monomeric or the aggregated form, have a tendency to integrate at the membrane interface.

**Figure 4:**
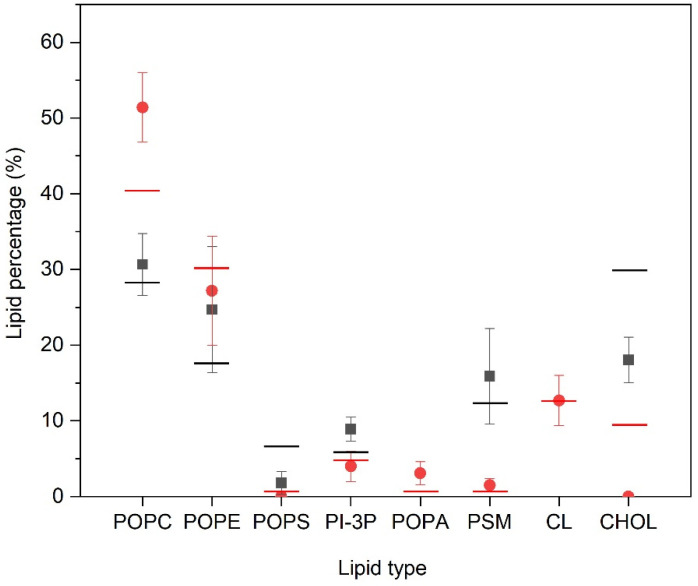
Relative percentage of the lipid molecules surrounding the PC1 molecules for the PM-containing systems (■) and the MIT-containing systems (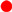) for the last 30 ns of the MD simulation. Lines correspond to the global percentage of each one of the lipids in the system (— for PM- and—for MIT-containing systems).

We have measured the number and type of lipid molecules that are located within two Å of the PC1 molecules, i.e., in close contact, and the results are shown in Figure 4. In the case of the PM-containing systems (systems one, two, and three) the most significant result is that CHOL is excluded from proximity to the PC1 molecules, since its global percentage composition is 30%, but reduces to about 18% when in contact with the PC1 molecules. The same can be said about POPS, since its global percentage is about 6.5%, which reduces to about 1.5% in the PM systems. On the contrary, PI-3P slightly increases, since its global percentage is 5.5% and in the surrounding area of the PC1 molecules it is about 9%. As for the other lipids, there is no clear preference for them to be near to the PC1 molecules, since the percentage of molecules near to the PC1 molecules is relatively similar to the global percentage in the system (Figure 4). For the MIT-containing systems, and similar to the PM-containing systems, the CHOL is completely excluded in the surrounding area of the PC1 molecules, since its global percentage composition is 9.5% but reduces to 0% in the surrounding area of the PC1 molecules (Figure 4). Interestingly, the quantity of the POPC molecules surrounding the PC1 molecules increased significantly, since its global percentage is 40% but increased to 51% around the PC1 molecules. POPA increased slightly, since its global percentage is about 0.5%, which increased to about 3% in the surrounding area of PC1 (Figure 4). For CL, the characteristic lipid of the mitochondrial membrane, there is no preference/aversion to being around the PC1 molecules, since there was no difference in the global and the local percentages. As for the other lipids, there is no clear preference for them to be near to the PC1 molecules, since the percentage of molecules near to PC1 molecules is relatively similar to the global percentage in the system (Figure 4). For all of the systems studied, the most significant result is that CHOL is the molecule that tends to be excluded from the surrounding area of the PC1 molecules.

It is known that molecules interacting with the membrane can affect the hydrocarbon chain order of the phospholipid acyl chains. Therefore, we have studied the effect of the PC1 molecules on the hydrocarbon chain order of the phospholipid acyl chains by obtaining their deuterium order parameter, S_CD_ (Supplementary Figures S10 and S11 for the PM and MIT systems, respectively). When there is complete disorder, the S_CD_ value is 0, and a value of 0.5 specifies full order along the normal bilayer [56]. The average –S_CD_ values of the acyl chains of all of the phospholipids in the PM model system, i.e., POPC, POPE, POPS, PI-3P, and PSM, were in agreement with the profiles that were observed earlier for the experimental and simulated data [36,57,58] (Supplementary Figure S10). However, for some of the phospholipids that were near to the PC1 molecules, there were significant, although not dramatic, changes in the S_CD_ profiles. For POPC, POPE, and PI-3P, a general decrease in the S_CD_ values was observed, whereas an increase was observed for POPS (Supplementary Figure S10). In contrast, no effect was observed for PSM. The decrease in S_CD_ that was observed for the phospholipids POPC, POPE, and PI-3P indicates that the PC1 molecules increase the fluidity of the hydrocarbon chains of these phospholipids, whereas the PC1 molecules increase the rigidity of the hydrocarbon chains of POPS. Similar to the PM system discussed above, the average –S_CD_ values of the acyl chains of all of the phospholipids in the MIT model system, i.e., POPC, POPE, POPS, POPA, PI-3P, CL, and PSM, were all in agreement with the profiles for the previous experimental and simulated data [36,57,58] (Supplementary Figure S11). However, for those phospholipids near to the PC1 molecules, there were significant changes in the S_CD_ profiles. In this case, and for all of the phospholipids, the presence of the PC1 molecules decreased the S_CD_ values, indicating an increase in the fluidity of the hydrocarbon chains. It can be inferred from these data that the PC1 molecules insert relatively well in between the hydrocarbon chains of the phospholipids. They do not show a dramatic effect on the anisotropy of the hydrocarbon chains, but the general trend is that the PC1 molecules increase the fluidity of the membrane, both in the PM and in the MIT systems.

## 4. Discussion

The bioactive procyanidins are known for their ample benefits to human health and have been shown to have a plethora of biological effects [3,7,8,9,10,11,12,13,14,15]. The bioactive effects of procyanidins in general, PC1 in particular, could be ascribed to their capability to localize in the biological membrane and to modulate the membrane structure and/or the dynamics, as well as their interaction with the lipids and proteins [23]. The numerous biological properties of these molecules, including procyanidins, demonstrate that diverse mechanisms are at play, but also show the presence of a common point of action. That common point is that the biological membrane and the bioactive properties of these molecules could be associated to a membrane modulation mechanism [59,60]. However, the biological mechanism of the procyanidins has been not identified, despite the knowledge that it could help in the development of new therapeutic molecules. Their biological, pharmacological, and medicinal properties should be linked to this membrane’s modulation effect, which itself rests on their structure, their interactions, their location, and their orientation in the membrane [59,60]. Therefore, the biological properties of PC1 could therefore be ascribed, not only to its possible interaction with proteins, but also to its ability to modulate the membrane’s biophysical properties.

**Figure 5:**
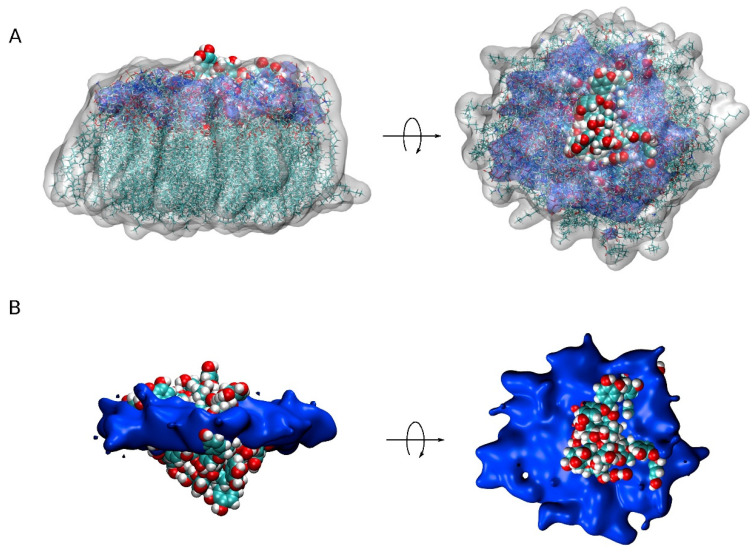
A view of all PC1 molecules in the monomer state together with the surrounding lipids (less than 7Å from PC1) for the five different systems (centered on PC1, 20 different superposed representations). (**A**) PC1 molecules in VDW representation, lipids in both licorice and white transparent surface representation and the phosphate atoms of the phospholipids in blue transparent surface representation. (**B**) PC1 molecules in VDW representation and the phosphate atoms of the phospholipids in blue opaque surface representation.

We have aimed to locate the molecule of PC1 and to determine the possible specific interactions of this molecule with the membrane lipids using MD simulations. For that objective, we have used two different model membranes, one mimicking the plasma membrane and the other mimicking the mitochondrial membrane. Our data reveal that PC1 tends to be located at the membrane interphase, with part of the molecule exposed to the water solvent and part of it reaching the first carbons of the hydrocarbon chains, but never extending beyond that so that they do not reach the middle of the bilayer leaflet (Figure 5, compare with Figure 3F). Furthermore, due to the specific three-dimensional structure of the PC1 molecule, it has no preferred orientation with all of its hydroxyl groups in the outer part of the molecule. One of the most interesting facts that we have found in this study is that the PC1 molecules completely exclude the molecule of CHOL, both in the PM and in the MIT membrane systems. In the case of the PM system, there were differences with the other lipids present, such as the reduction in POPS and an increase in PI-3P; however, no significant differences were found for the other lipids in the system. In the case of the MIT system, both POPC and POPA seem to increase in the surrounding area of the PC1 molecules. Similar to the PM system, no differences were found for the other lipids in the MIT system. Yet, one of the most remarkable facts about PC1 is that, in the solution, it has a tendency to combine forming dimers, trimers, and higher-order aggregates between the different PC1 molecules. These groups of PC1 molecules form spontaneously through the formation of hydrogen bonds; the formation of the hydrogen bonds being independent of the systems studied here, be it either the PM or the MIT system. Interestingly, the formation of the aggregates did not prevent the PC1 molecules from interacting with the membrane, either in the PM or the MIT systems. PC1 is known to be stable under gastric conditions, it is not fragmented into its monomer constituents, it is found in the plasma after ingestion and, fundamentally, relatively high concentrations and high frequency treatments of PC1 have no systemic toxicities. However, the formation of the PC1 aggregates could hamper its bioactive properties and, consequently, this should be taken into account when planning its use in clinical trials in order to choose an appropriate vehicle for its preparation. Our work should help to advance these molecules as therapeutic molecules by opening up new avenues for future medical advances.

## 5. Conclusions

PC1 tends to be located at the membrane interphase, with a part of the molecule exposed to the solvent and part of it reaching the carbonyl region of the hydrocarbon chains. PC1 molecules, when inserted into the membrane, have no preferred orientation, having all of the hydroxyl groups in the outer part of the molecule. Significantly, PC1 completely excludes the molecule of CHOL in both the PM and the MIT systems, so that the existence of domains with and without cholesterol cannot be ruled out. Outstandingly, PC1 molecules have a tendency to spontaneously associate, forming aggregates, which does not prevent them from interacting with the membrane.

## Figures and Tables

**Table 1:** Systems and number of components used in this study. The NaCl concentration was 0.15 M. The production trajectories for each one of the five different systems were calculated for 675 ns.

	*Plasma* *Membrane Model*			*Mitochondrial Membrane Model*	
	*SYSTEM 1*	*SYSTEM 2*	*SYSTEM 3*	*SYSTEM 4*	*SYSTEM 5*
**POPC**	80	80	80	90	90
**POPE**	48	48	48	68	68
**PI-3P**	16	16	16	10	10
**POPS**	18	18	18	2	2
**POPA**	-	-	-	2	2
**PSM**	34	34	34	2	2
**CL**	-	-	-	28	28
**CHOL**	84	84	84	20	20
***Total***	280	280	280	222	222
**PC1**	16	8	4	8	16
**H_2_O**	27,914	28,420	28,922	33,598	33,136
**Na^+^**	145	146	148	176	172
**Cl^−^**	79	80	82	96	94
**Dimensions** **x/y/z (Å)**	104/104/120			112/112/124	

**Table 2:** Number of oligomer types and number of molecules forming them (in parenthesis) for the five systems studied in this work *.

	Number of					
	Monomer	Dimer	Trimer	Tetramer	Oligomer	PC1 (Total)
**SYSTEM 1** PM	2 (2)	2 (4)	-	-	1 (10)	*16*
**SYSTEM 2** PM	5 (5)	-	1 (3)	--	-	*8*
**SYSTEM 3** PM	4 (4)	-	-	-	-	*4*
**SYSTEM 4** MIT	6 (6)	1 (2)	-	-	-	*8*
**SYSTEM 5** MIT	4 (4)	-	-	1 (4)	1 (8)	*16*
	*21*	*6*	*3*	*4*	*18*	*52*

* PM: plasma membrane, MIT: mitochondrial membrane.

## Data Availability

Not applicable.

## Supplementary Materials

The following supporting information can be downloaded at: https://www.mdpi.com/article/10.3390/membranes12070692/s1, Figure S1: Chemical structures of the lipid molecules used in this study.(A) POPC (1-palmitoyl-2-oleoyl-sn-glycero-3-phosphatidylcholine), (B) POPE (1-palmitoyl-2-oleoyl-sn-glycero-3-phosphatidylethanolamine), (C) POPS (1-palmitoyl-2-oleoyl-sn-glycero-3-phosphoserine), (D) POPA (1-Palmitoyl-2-oleoyl-sn-glycero-3-phosphate), (E) PI-3P (1-palmitoyl-2-oleoyl-sn-glycero-3-phosphoinositol-3-phosphate), (F) CL (1′,3′-bis[1-palmitoyl-2-oleoyl-sn-glycero-3-phospho]-glycerol), (G) PSM (N-stearoyl-D-erythro-sphingosylphosphorylcholine) and (H) CHOL (cholesterol); Figure S2: Time variation of the molecular areas for the whole simulation time for each lipid species in the (A) system 1, (B) system 2, (C) system 3, (D) system 4 and (E) system 5 membrane model systems. The following lipids are depicted: POPC (black), POPE (blue), POPS (red), PI-3P (magenta), POPA (orange), PSM (olive), CL (violet) and CHOL (wine); Figure S3: Time variation of (A) membrane thickness for the whole simulation time and (B) average membrane thickness for the last 30 n of simulation for system 1 (black), system 2 (blue), system 3 (red), system 4 (magenta) and system 5 (olive); Figure S4: Average COM z-axis for the last 30 n of simulation for all the PC1 molecules in system 1. Blue and black histograms correspond to monomer and oligomer states, respectively. The average COM of the phosphate atoms of the phospholipids is depicted in red color; Figure S5: Average COM z-axis for the last 30 n of simulation for all the PC1 molecules in system 2. Blue and black histograms correspond to monomer and oligomer states, respectively. The average COM of the phosphate atoms of the phospholipids is depicted in red col-our; Figure S6: Average COM z-axis for the last 30 n of simulation for all the PC1 molecules in system 3. Blue and black histograms correspond to monomer and oligomer states, respectively. The average COM of the phosphate atoms of the phospholipids is depicted in red color; Figure S7: Average COM z-axis for the last 30 n of simulation for all the PC1 molecules in system 4. Blue and black histograms correspond to monomer and oligomer states, respectively. The average COM of the phosphate atoms of the phospholipids is depicted in red color; Figure S8: Average COM z-axis for the last 30 n of simulation for all the PC1 molecules in system 5. Blue and black histograms correspond to monomer and oligomer states, respectively. The average COM of the phosphate atoms of the phospholipids is depicted in red color; Figure S9: Mass density profiles for the last 30 ns of the MD simulation for (A, B) system 1, (C, D) system 2, (E, F) system 3, (G, H) system 4 and (I, J) system 5. The global mass density of all the lipids and all the PC1 molecules are shown in (A, C, E, G and I), whereas the mass density of the phosphate atoms of the phospholipids and each one of the individual PC1 molecules are shown in (B, D, F, H and J). The mass density profiles correspond to POPC (blue), POPE (orange), POPS (red), PI-3P (magenta), POPA (wine), PSM (olive), CL (navy), CHOL (violet) and PC1 (black). In (B, D, F, H and J) the PC1 molecules in the monomer state are depicted as dotted red lines; Figure S10: Average deuterium order parameter –SCD calculated for the hydrocarbon chains of the phospholipids in the PM system. (A, C, E, G) oleoyl and (B, D, F, H) palmitoyl acyl chains of (A, B) POPC, (C, D) POPE, (E, F) POPS and (G, H) PI-3P as well as the palmitoyl (I) and sphyngosyl (J) acyl chains of PSM. The data correspond to the bulk phospholipid acyl chains (-■-) and the phospholipid acyl chains within 5 Å of the PC1 molecules (--). The analysis was carried out for the last 30 ns of simulation; Figure S11: Average deuterium order parameter –SCD calculated for the hydrocarbon chains of the phospholipids in the MIT system. (A, C, E, G, I) oleoyl and (B, D, F, H, J) palmitoyl acyl chains of (A, B) POPC, (C, D) POPE, (E, F) POPS, (G, H) PI-3P and (K, L) POPA, the palmitoyl (K) and vacenoyl (L) acyl chains of CL and the palmitoyl (M) and sphyngosyl (N) acyl chains of PSM. The data corre-spond to the bulk phospholipid acyl chains (-■-) and the phospholipid acyl chains within 5 Å of the PC1 molecules (--). The analysis was carried out for the last 30 ns of simulation; Table S1: Average molecular area (Å2) and membrane thickness (Å) for the last 30 ns of simulation of all the lipids in the systems studied in this work.

## Abbreviations

CHOL	Cholesterol
CL	(1′,3′-bis[1-palmitoyl-2-oleoyl-sn-glycero-3-phospho]-glycerol)
COM	Center of mass
MIT	Mitochondrial membrane
PC1	Procyanidin C1 [(-)-epicatechin–(4β→8)-(-)-epicatechin (4β→8)-(-)-epicatechin]
PI-3P	1-Palmitoyl-2-oleoyl-sn-glycero-3-phosphoinositol-3-phosphate
PM	Plasma membrane
POPA	1-Palmitoyl-2-oleoyl-sn-glycero-3-phosphate
POPC	1-Palmitoyl-2-oleoyl-sn-glycero-3-phosphocholine
POPE	1-Palmitoyl-2-oleoyl-sn-glycero-3-phosphoethanolamine
POPS	1-Palmitoyl-2-oleoyl-sn-glycero-3-phospho-L-serine
PSM	N-Palmitoyl-D-erythro-sphingosylphosphorylcholine
