# Supplementary material for: Procyanidin C1 Location, Interaction, and Aggregation in Two Complex Biomembranes
Source: Membranes (Basel). 2022 Jul 5;12(7):692. doi: 10.3390/membranes12070692 (PMC9319219; doi:10.3390/membranes12070692)
Supplement: Supplementary file 1 [file membranes-12-00692-s001.zip › membranes-1804604-supplementary.pdf]

Supplementary Material

Procyanidin C1 Location, Interaction, and Aggregation in two Complex Biomembranes

José Villalaín

Institute of Research, Development, and Innovation in Healthcare Biotechnology (IDiBE), Universidad Miguel Hernández, E-03202 Elche-Alicante, Spain; jvillalain@umh.es; Tel: +34-966-658-762; Fax: +34-966-658-758

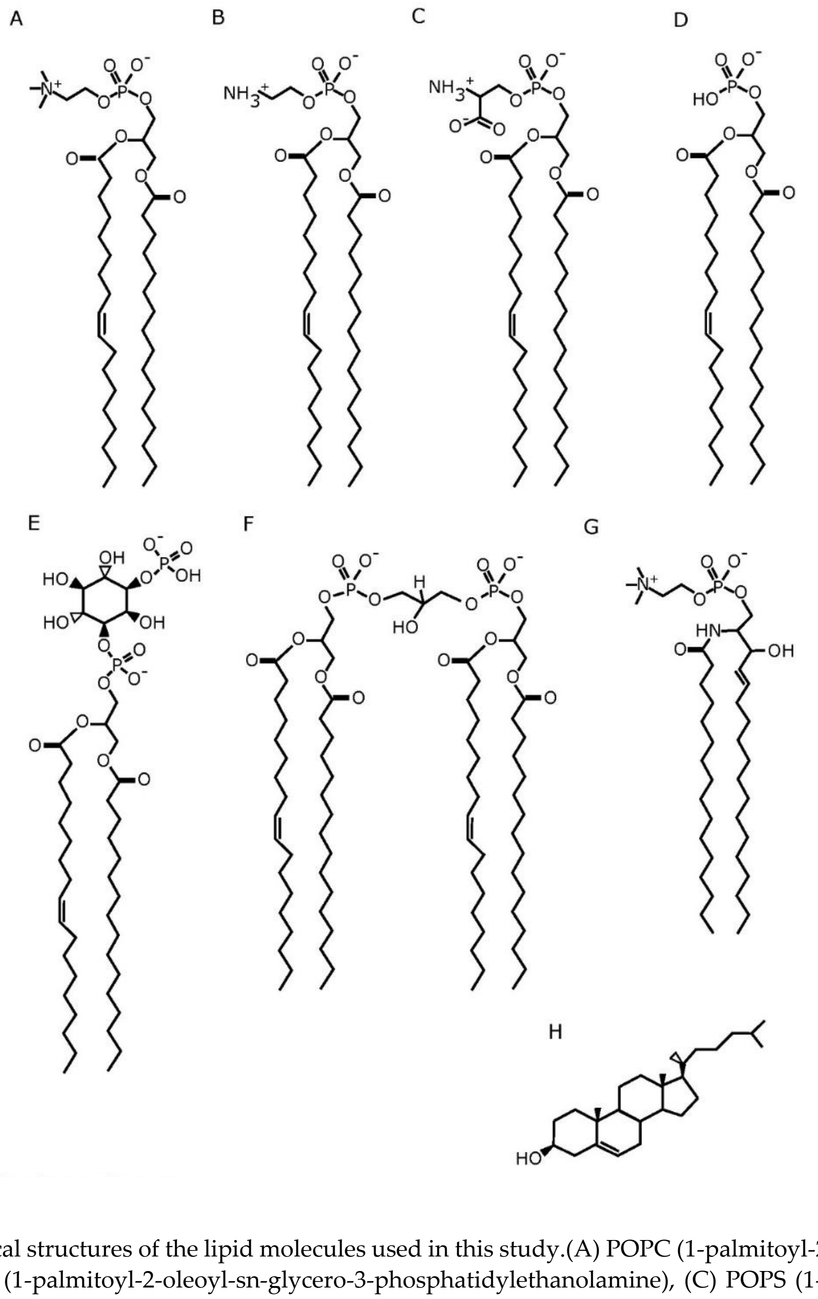

Supplementary Figure S1. Chemical structures of the lipid molecules used in this study.(A) POPC (1-palmitoyl-2-oleoyl-sn-glycero-3-phosphatidylcholine), (B) POPE (1-palmitoyl-2-oleoyl-sn-glycero-3-phosphatidylethanolamine), (C) POPS (1-palmitoyl-2-oleoyl-sn-glycero-3-phosphoserine), (D) POPA (1-Palmitoyl-2-oleoyl-sn-glycero-3-phosphate), (E) PI-3P (1-palmitoyl-2-oleoyl-sn-glycero-3-phosphoinositol-3-phosphate), (F) CL (1',3'-bis[1-palmitoyl-2-oleoyl-sn-glycero-3-phospho]-glycerol), (G) PSM (N-stearoyl-D-erythro-sphingosylphosphorylcholine) and (H) CHOL (cholesterol).

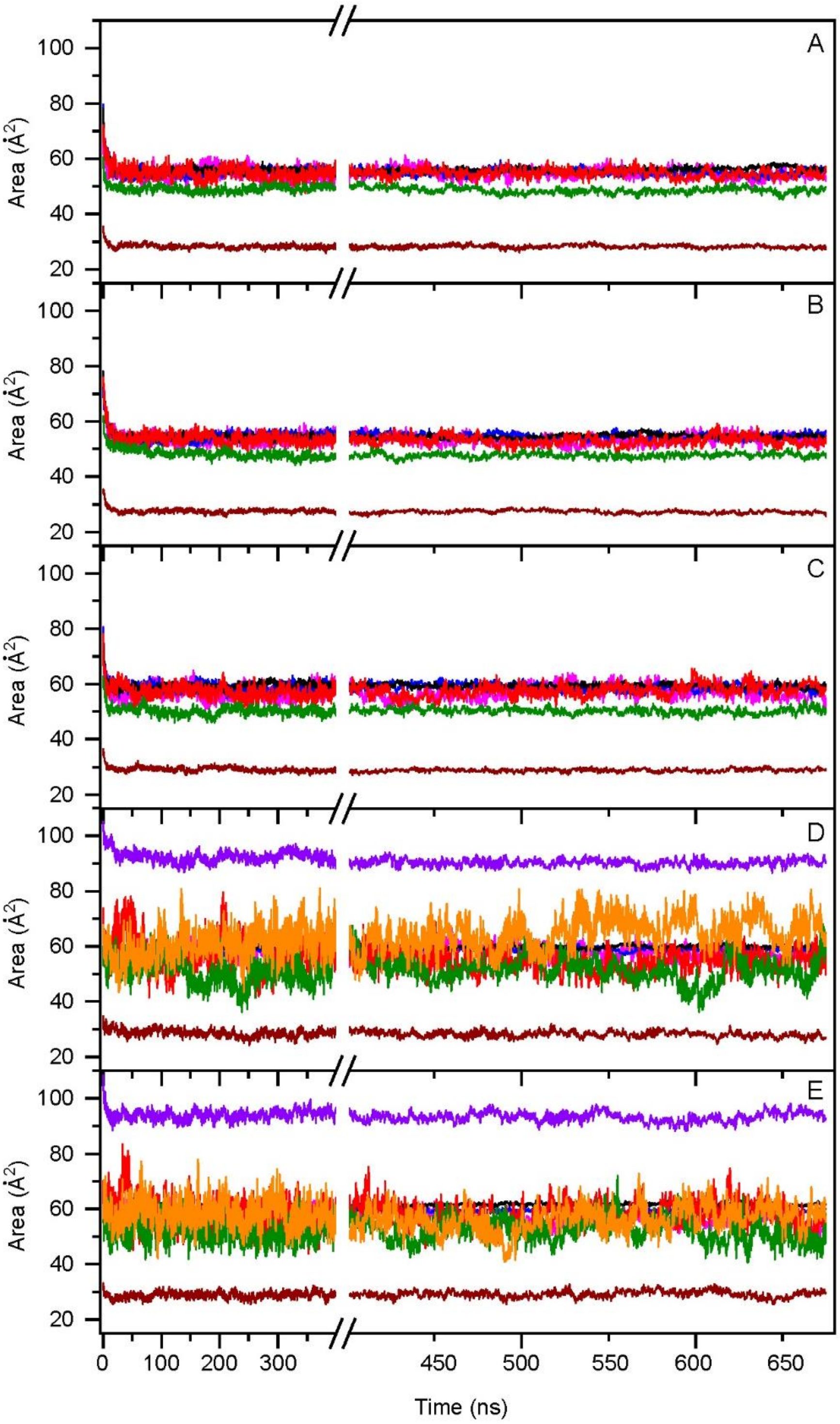

Supplementary Figure S2. Time variation of the molecular areas for the whole simulation time for each lipid species in the (A) system 1, (B) system 2, (C) system 3, (D) system 4 and (E) system 5 membrane model systems. The following lipids are depicted: POPC (black), POPE (blue), POPS (red), PI-3P (magenta), POPA (orange), PSM (olive), CL (violet) and CHOL (wine).

Supplementary Table S1. Average molecular area (Å²) and membrane thickness (Å) for the last 30 ns of simulation of all the lipids in the systems studied in this work.

.

	AREA LIPIDS (Å²)							
**SYSTEMS**	**POPC**	**POPE**	**POPS**	**PI-3P**	**PSM**	**POPA**	CL	CHOL
**S1**	56.7±0.9	55.2±0.9	54.4±1.4	54.2±1.3	48.2±1.1	-	-	27.9±0.4
**S2**	54.4±1.2	55.3±0.7	54.3±1.2	53.7±1.5	47.9±0.8	-	-	27.1±0.4
**S3**	59.2±0.8	58.6±1.1	58.5±1.8	55.9±1.7	50.8±1.1	-	-	28.9±0.6
**S4**	59.9±0.5	59.1±0.6	55.4±3.4	56.4±1.5	51.9±5.1	65.3±5.4	90.8±1.2	27.4±0.9
**S5**	59.8±0.8	59.5±0.7	55.9±3.6	55.3±2.6	48.9±3.4	57.7±4.1	94.6±1.4	28.9±1.2

	MEMBRANE THICKNESS (Å)			
**S1**	**S2**	**S3**	**S4**	**S5**
43.9±0.1	44.3±0.2	43.2±0.2	43.3±0.1	42.7±0.2

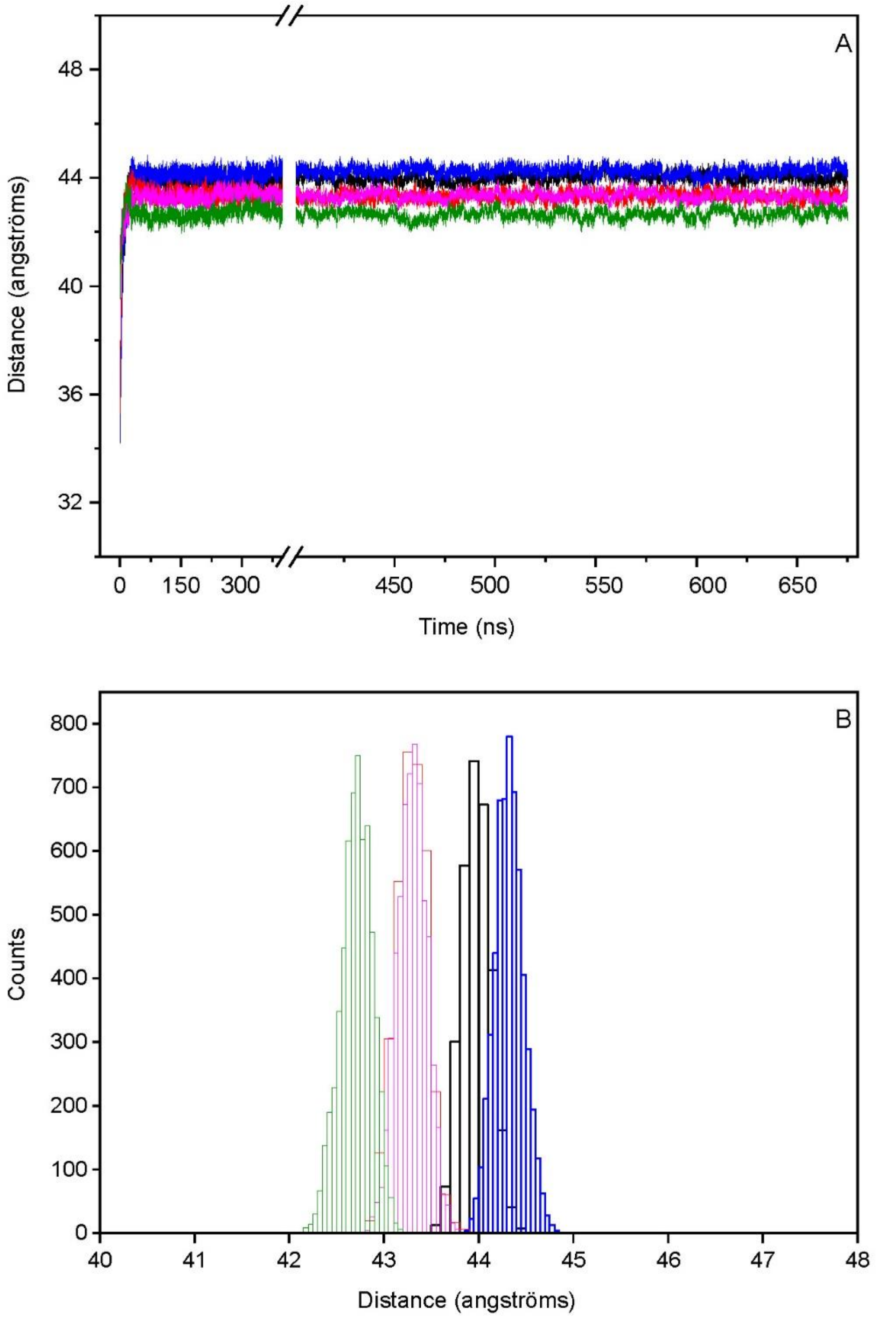

Supplementary Figure S3. Time variation of (A) membrane thickness for the whole simulation time and (B) average membrane thickness for the last 30 n of simulation for system 1 (black), system 2 (blue), system 3 (red), system 4 (magenta) and system 5 (olive).

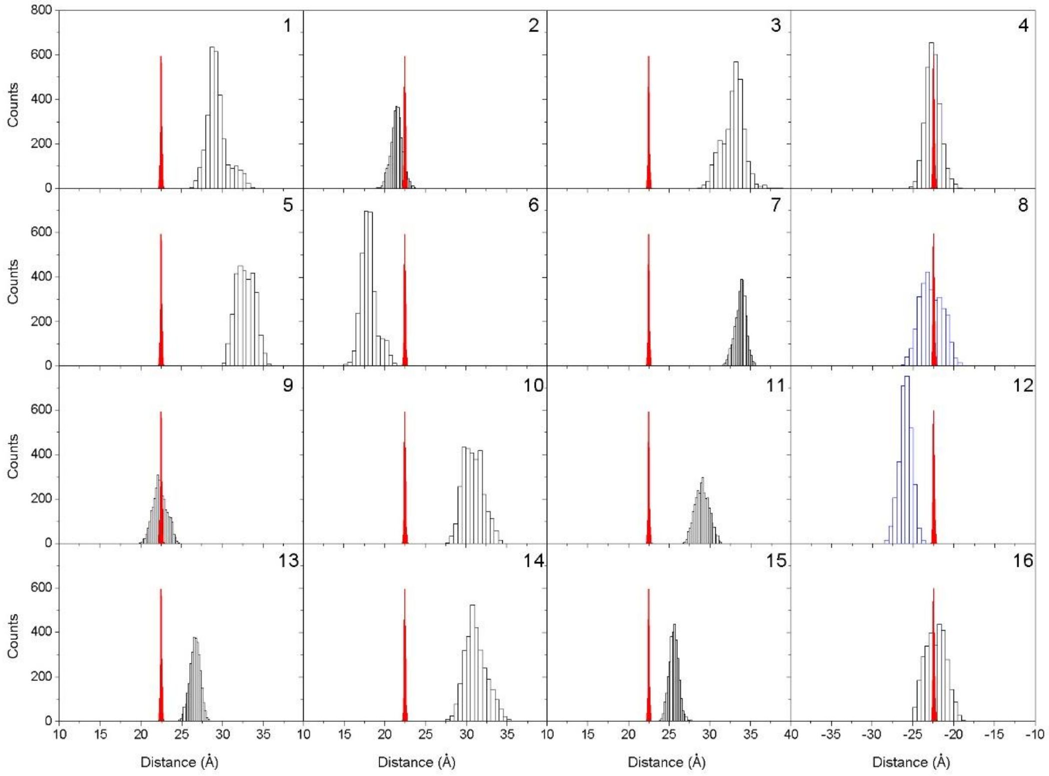

Supplementary Figure S4. Average COM z-axis for the last 30 n of simulation for all the PC1 molecules in system 1. Blue and black histograms correspond to monomer and oligomer states, respectively. The average COM of the phosphate atoms of the phospholipids is depicted in red colour.

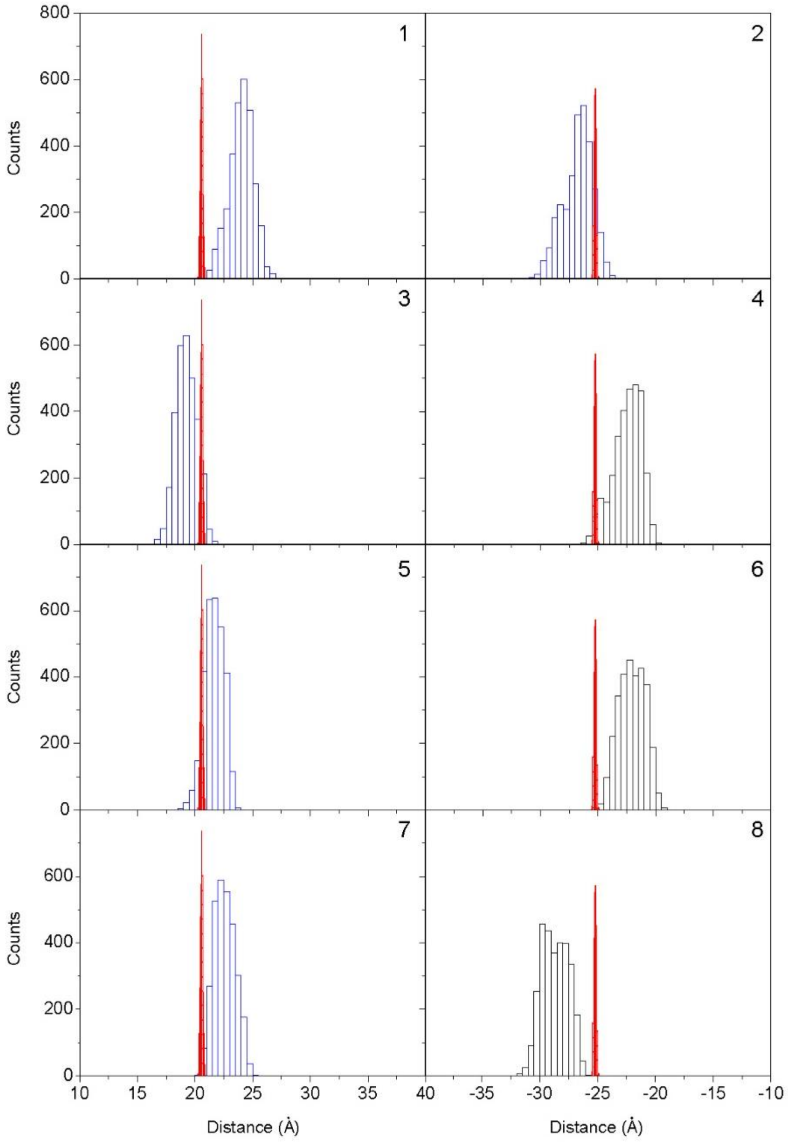

Supplementary Figure S5. Average COM z-axis for the last 30 n of simulation for all the PC1 molecules in system 2. Blue and black histograms correspond to monomer and oligomer states, respectively. The average COM of the phosphate atoms of the phospholipids is depicted in red colour.

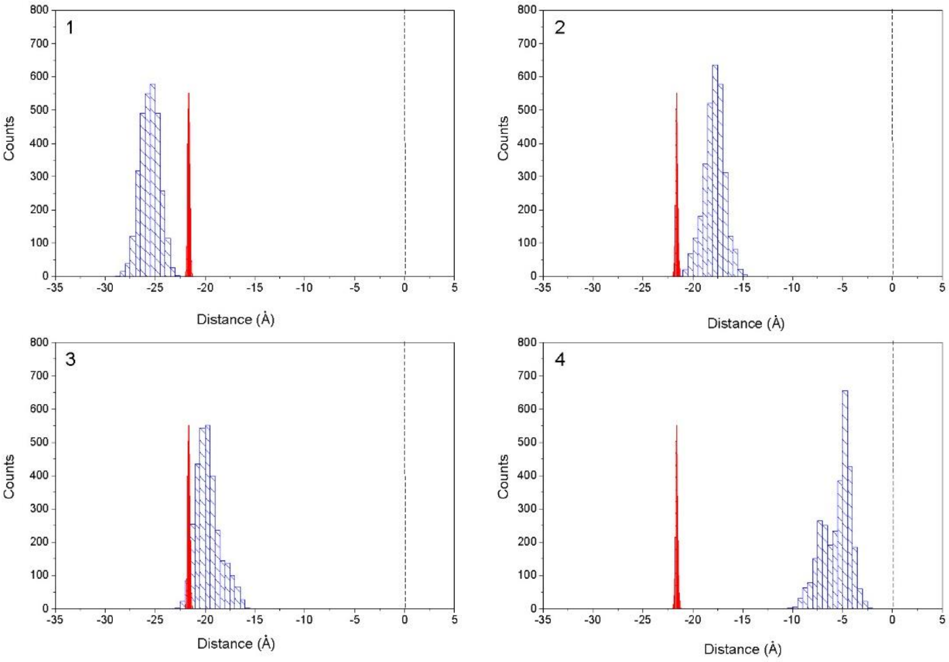

Supplementary Figure S6. Average COM z-axis for the last 30 n of simulation for all the PC1 molecules in system 3. Blue and black histograms correspond to monomer and oligomer states, respectively. The average COM of the phosphate atoms of the phospholipids is depicted in red colour.

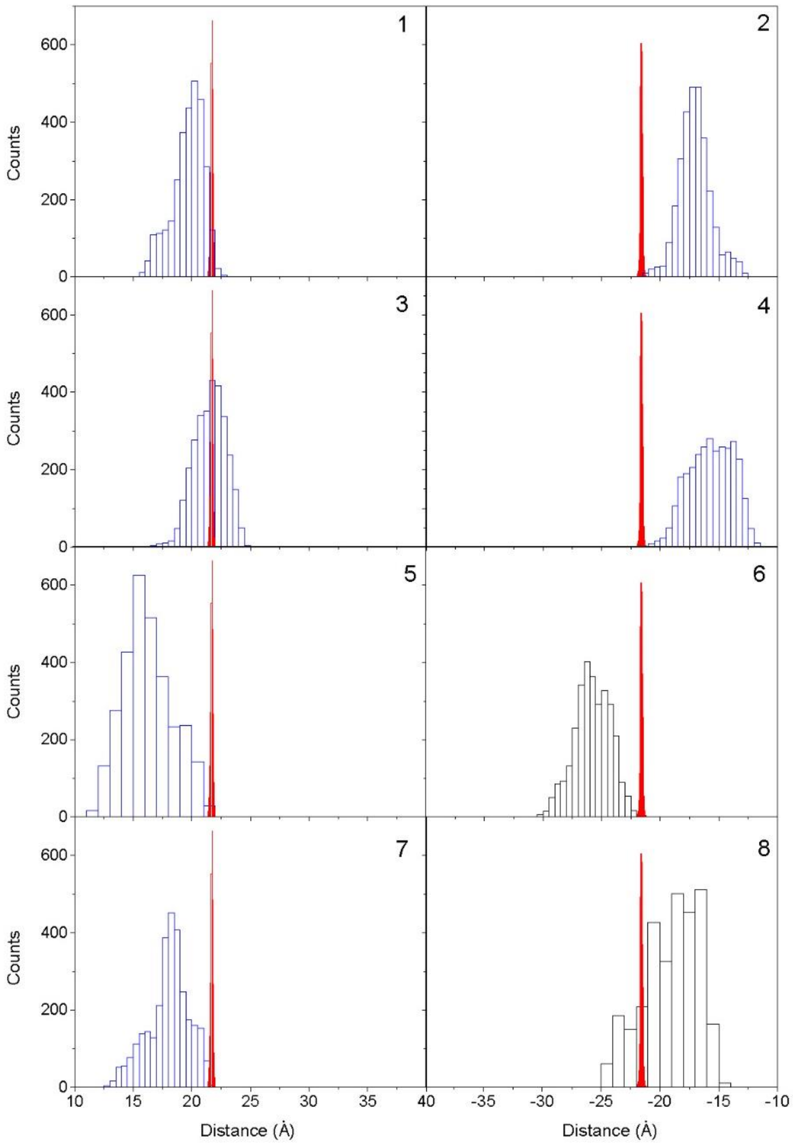

Supplementary Figure S7. Average COM z-axis for the last 30 n of simulation for all the PC1 molecules in system 4. Blue and black histograms correspond to monomer and oligomer states, respectively. The average COM of the phosphate atoms of the phospholipids is depicted in red colour.

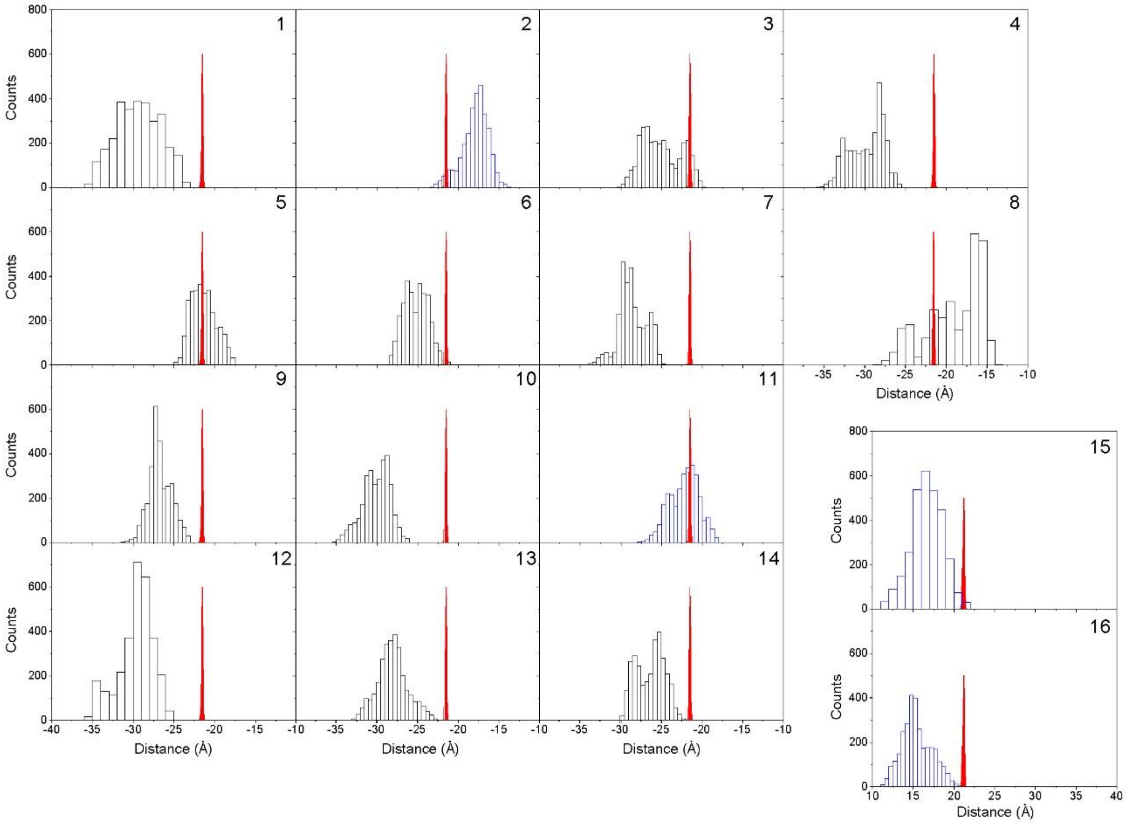

Supplementary Figure S8. Average COM z-axis for the last 30 n of simulation for all the PC1 molecules in system 5. Blue and black histograms correspond to monomer and oligomer states, respectively. The average COM of the phosphate atoms of the phospholipids is depicted in red colour.

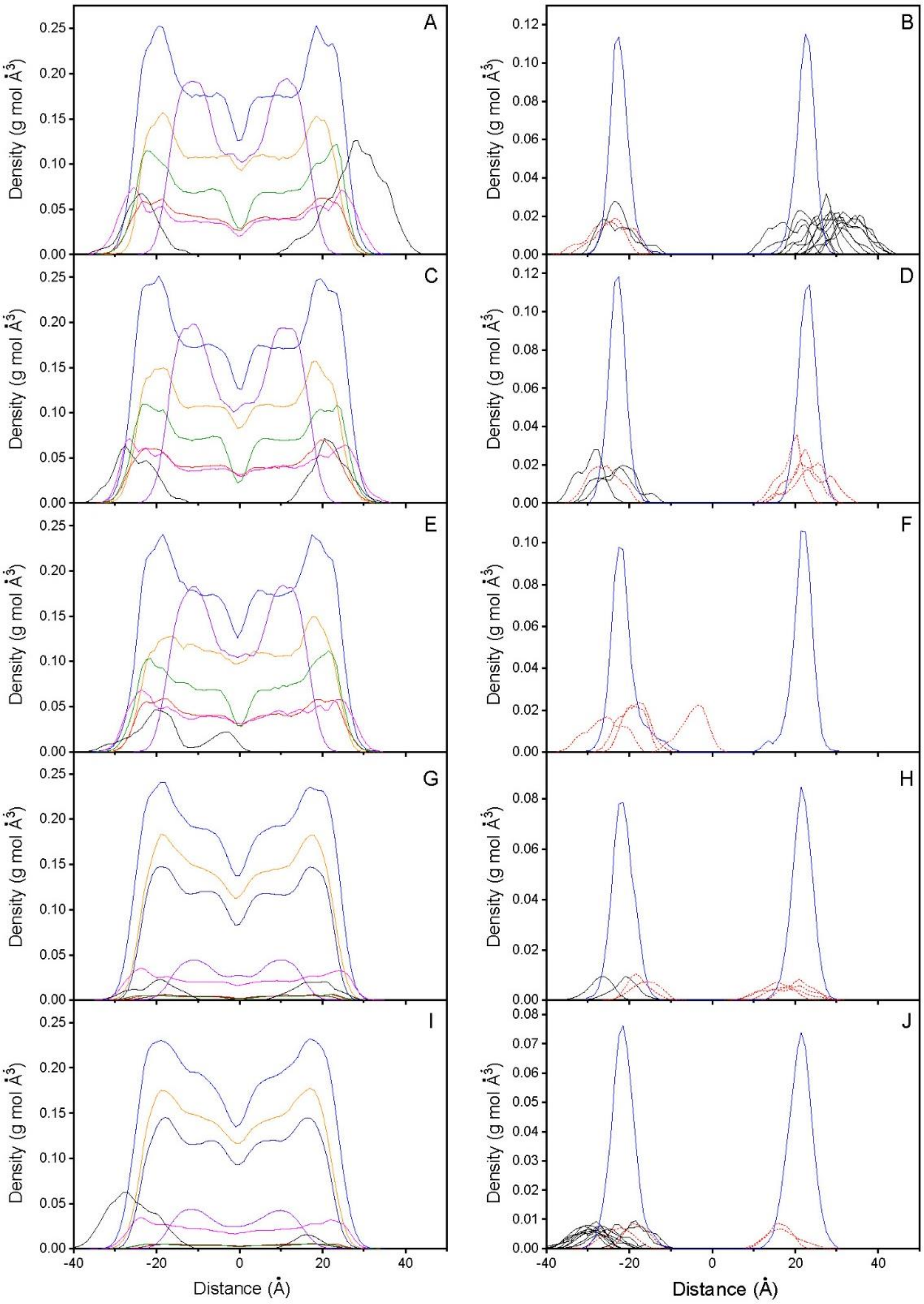

Supplementary Figure S9. Mass density profiles for the last 30 ns of the MD simulation for (A,B) system 1, (C,D) system 2, (E,F) system 3, (G,H) system 4 and (I,J) system 5. The global mass density of all the lipids and all the PC1 molecules are shown in (A,C,E,G and I), whereas the mass density of the phosphate atoms of the phospholipids and each one of the individual PC1 molecules are shown in (B,D,F,H and J). The mass density profiles correspond to POPC (blue), POPE (orange), POPS (red), PI-3P (magenta), POPA (wine), PSM (olive), CL (navy), CHOL (violet) and PC1 (black). In (B,D,F,H and J) the PC1 molecules in the monomer state are depicted as dotted red lines.

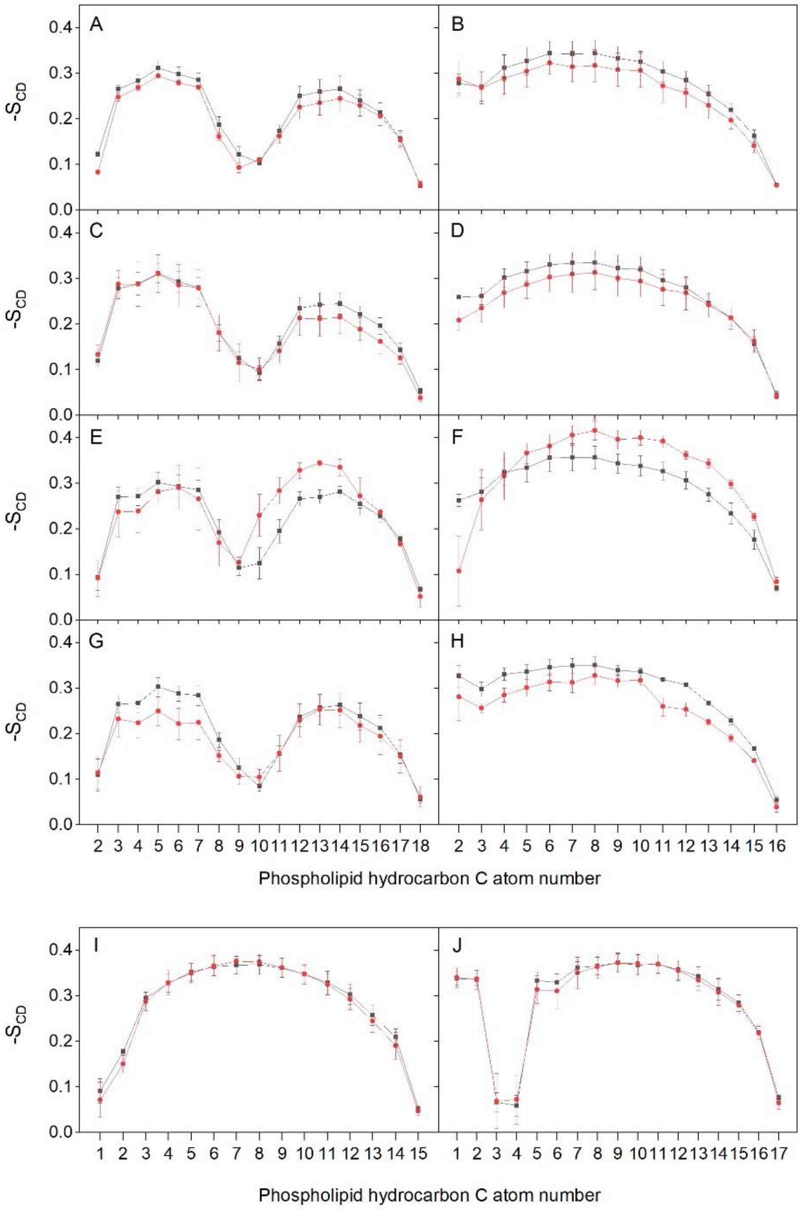

Supplementary Figure S10. Average deuterium order parameter –S_{CD} calculated for the hydrocarbon chains of the phospholipids in the PM system. (A, C, E, G) oleoyl and (B, D, F, H) palmitoyl acyl chains of (A, B) POPC, (C, D) POPE, (E, F) POPS and (G,H) PI-3P as well as the palmitoyl (I) and sphyngosyl (J) acyl chains of PSM. The data correspond to the bulk phospholipid acyl chains (-■-) and the phospholipid acyl chains within 5 Å of the PC1 molecules (-●-). The analysis was carried out for the last 30 ns of simulation.

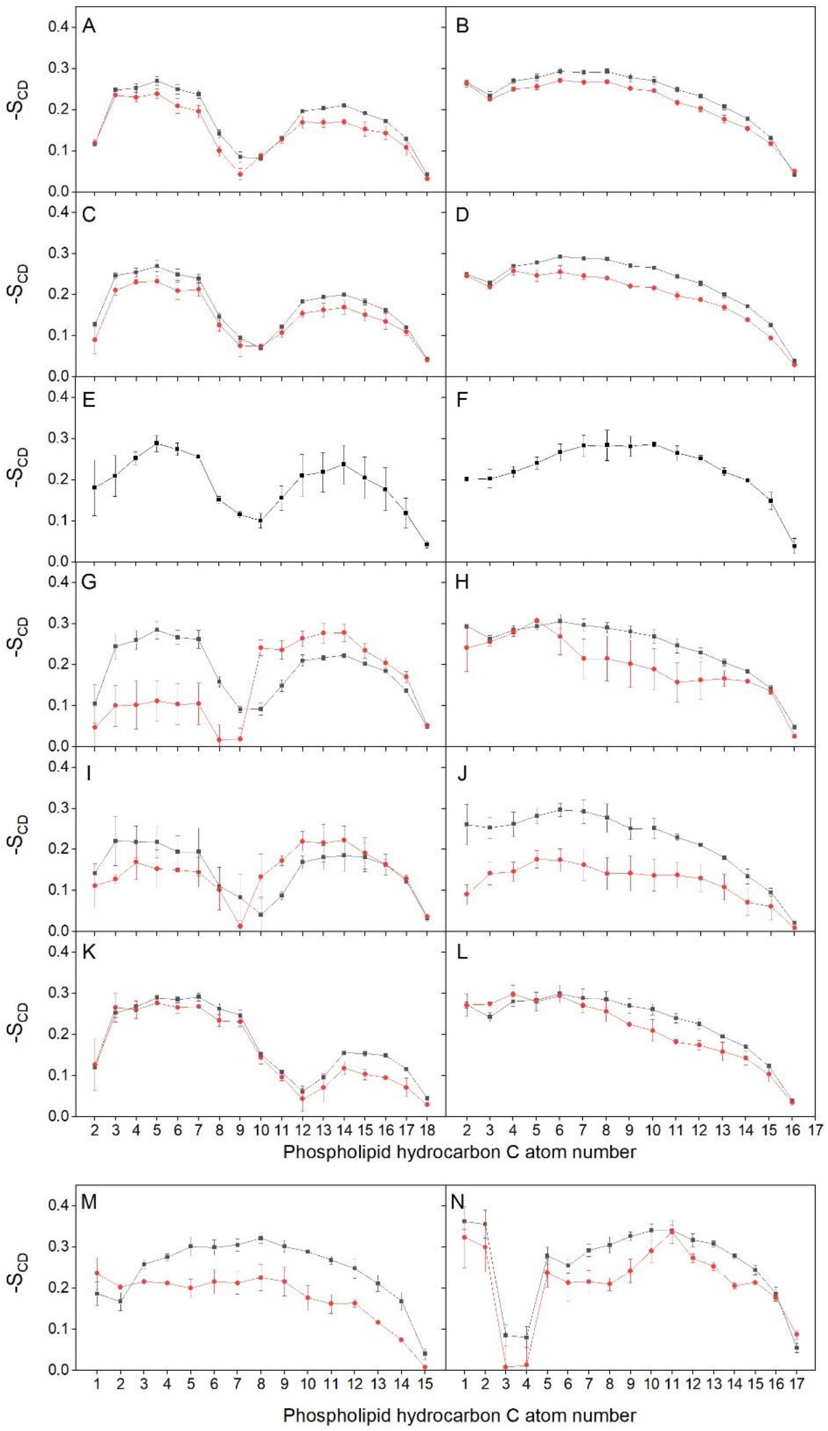

Supplementary Figure S11. Average deuterium order parameter $-S_{CD}$ calculated for the hydrocarbon chains of the phospholipids in the MIT system. (A, C, E, G, I) oleoyl and (B, D, F, H, J) palmitoyl acyl chains of (A, B) POPC, (C, D) POPE, (E, F) POPS, (G, H) PI-3P and (K, L) POPA, the palmitoyl (K) and vacenoyl (L) acyl chains of CL and the palmitoyl (M) and sphyngosyl (N) acyl chains of PSM. The data correspond to the bulk phospholipid acyl chains (-■-) and the phospholipid acyl chains within 5 Å of the PC1 molecules (-●-). The analysis was carried out for the last 30 ns of simulation.
